# Ribavirin restores ESR1 gene expression and tamoxifen sensitivity in ESR1 negative breast cancer cell lines

**DOI:** 10.1186/1868-7083-3-8

**Published:** 2011-12-05

**Authors:** Anne Sappok, Ulrich Mahlknecht

**Affiliations:** 1 Saarland University Medical Center, Department of Internal Medicine, Division of Immunotherapy and Gene Therapy, Homburg/Saar, Germany

**Keywords:** epigenetic, estrogen receptor alpha, HDAC, methylation, ribavirin, SAHA

## Abstract

Tumor growth is estrogen independent in approximately one-third of all breast cancers, which makes these patients unresponsive to hormonal treatment. This unresponsiveness to hormonal treatment may be explained through the absence of the estrogen receptor alpha (ESR1). The ESR1 gene re-expression through epigenetic modulators such as DNA methyltransferase inhibitors and/or histone deacetylase inhibitors restores tamoxifen sensitivity in ESR1 negative breast cancer cell lines and opens new treatment horizons in patients who were previously associated with a poor prognosis.

In the study presented herein, we tested the ability of ribavirin, which shares some structural similarities with the DNA-methyltransferase inhibitor 5-azacytidine and which is widely known as an anti-viral agent in the treatment of hepatitis C, to restore ESR1 gene re-expression in ESR1 negative breast cancer cell lines.

In our study we identified ribavirin to restore ESR1 gene re-expression alone and even more in combination with suberoylanilide hydroxamic acid (SAHA - up to 276 fold induction).

Ribavirin and analogs could pave the way to novel translational research projects that aim to restore ESR1 gene re-expression and thus the susceptibility to tamoxifen-based endocrine treatment strategies.

## Introduction

Breast cancer is the most frequent type of cancer in women in the Western world and the second leading cause of cancer death. Approximately one in 8 women living in the USA today is being diagnosed with breast cancer at some point during her lifetime [1].

In the clinic, the estrogen receptor (ESR) and more precisely the estrogen receptor α (ESR1) is an important prognostic disease marker [2]. Approximately two-thirds of breast cancers are ESR1-positive.

The binding of estrogen to the ESR1 is not only a key regulator for the physiological growth and differentiation of the mammary gland, it is also a key element in the malignant progression of breast cancer, i.e. the growth of ESR1 expressed breast cancer cells is stimulated by estrogen, which in turn makes it accessible to endocrine treatment strategies, while breast cancers that do not express ESR1 exhibit a primary resistance to endocrine treatment [3,4]. Therefore, the presence of ESR1 correlates with increased disease-free survival and a better prognosis when compared to ESR1-negative breast cancers [5]. While at the time of diagnosis up to one-third of breast cancers are ESR1 negative, quite a few cancers that are initially ESR1 positive lose the ESR1 during the course of tumor progression and are therefore no longer responsive to endocrine therapy designed to block ESR1 function [6].

While the lack of ESR1 expression appears to be caused by genetic mutations in only less than 1% of ESR1-negative cancers, there is increasing evidence that epigenetic alterations of cytosine residues at the level of the ESR1 promoter DNA and the posttranslational modification of N-terminal ends of histone proteins are responsible for the absence of ESR1 expression in ESR1-negative cancers [7,8]. Typically, *hyper*methylation of CpG elements in the 5' regulatory region of the ER gene is associated with loss of ESR gene expression in ESR-negative breast cancers [9].

5-aza-2'-deoxycytidine, which is a cytidine analog leads to partial demethylation of the ESR promoter and consequently to re-expression of ESR mRNA and synthesis of functional ESR protein [10]. 5-aza-2'-deoxycytidine directly and irreversibly binds the DNA methyltransferase (DNMT), which blocks the DNMT methylating activity. 5-aza-2'-deoxycytidine is being incorporated into DNA in the presence of S-adenosylmethionine (SAM), which is a donor of methyl groups [11].

In addition to the hypomethylation of CpG elements within the ESR promoter, gene silencing may also be mediated through the posttranslational deacetylation of histone proteins, which goes along with a condensation of the chromatin architecture and therefore the silencing of associated genes. This inactive, highly condensed chromatin architecture is structured around hypermethylated ESR promoter CpG clusters. Methyl CpG-binding proteins recruit histone deacetylase (HDAC) enzymes which consequently deacetylate specific lysine groups on N-terminal histone ends, preferentially on H3 and H4 histone proteins. This results in a condensed nucleosome structure on the basis of an ionic interaction between positively charged lysine residues and the negatively charged DNA that limits transcription [12-14].

*In vitro *studies identified the HDAC inhibitor Trichostatin A (TSA) to restore functional ESR1 mRNA and protein expression in ESR1 negative breast cancer cells [13]. Also, suberoylanilide hydroxamic acid (SAHA), an HDAC inhibitor that is used in the clinic for various indications (e.g. cutaneous T-cell lymphoma), was able to re-express ESR1 in addition to its suppressive effects on the epidermal growth factor signaling pathway [15]. The combination of a DNMT inhibitor (e.g. 5-aza-2'-deoxycytidine) with an HDAC inhibitor such as TSA goes along with the synergistic reactivation of functional ESR1 [8]. The re-expression of ESR1 is by far more effective for the combination of agents when compared to the single agent treatment with 5-aza-2'-deoxycytidine or TSA.

Another way to reduce the degree of genomic DNA methylation is based on the inhibition of the S-adenosylhomocysteine (SAH) hydrolase. SAH, which is the reactant of the SAH hydrolase, is the demethylated form of SAM, the methyl donor of DNMT, which on the other hand, is a powerful inhibitor of methyltrasferase enzymes through its competing activity for the SAM binding site. SAH is further hydrolyzed by SAH hydrolase to adenosine and homocysteine [16]. The inhibition of theses enzymes results in increased levels of SAH, which in turn, inhibits the DNMT enzymes [17].

In the study presented herein, we report ribavirin to induce the re-expression of ESR1. Ribavirin inhibits the inosinate dehydrogenase and is a synthetic oral nucleoside analog, that is known since long as an antiviral agent in the treatment of a broad spectrum of DNA and RNA viruses, particularly in combination with alpha interferon for the treatment of chronic hepatitis C [18]. In addition to other effects, ribavirin also inhibits the SAH hydrolase, which may in fact explain the hypomethylation of the ESR promoter DNA and the associated re-expression of ESR1 [19]. Also, the chemical structure of ribavirin is highly similar to the chemical structure of 5-azycytidine, which inhibits the DNMT and could therefore also help to explain how hypomethylation of the ESR promoter DNA is being associated with the re-expression of ESR1 [20].

We show that ribavirin induces the re-expression of functional ESR1 in the ESR1-negative cells MDA-MB-231. This effect is strongly potentiated for the combination of ribavirin with an HDAC inhibitor, which was - in turn - able to restore the response to tamoxifen treatment.

Taken together, our data show that the combination of ribavirin and SAHA induces the re-expression of functional ESR1 and restores sensitivity of ESR1-negative cells to endocrine therapies.

## Materials and methods

### Cell Culture and Treatment Protocols

The human breast cancer cell lines MDA-MB-231, SK-BR-3 and UACC3199 were all cultured in DMEM. The medium was supplemented with 10% FBS and 1% penicillin/streptomycin. The cells were grown at 37°C and 5% CO_2_.

12 h prior to treatment the cells were plated at a density of 500,000 cells/100 mm tissue culture dishes. After 12 h, the culture medium was changed and the cells were treated with the reagents as indicated below. Ribavirin (Sigma-Aldrich, Munich, Germany) was used at concentrations of 9, 90 and 900 μM, respectively for up to 8 days. Trichostatin A (TSA, 0.33 μM) (Sigma-Aldrich, Munich, Germany) or suberoylanilide hydroxamic acid (SAHA, 10 μM) (Sigma-Aldrich, Munich, Germany) were only added during the last 12 h. For the treatment with 4-OH Tamoxifen (Sigma-Aldrich, Munich, Germany) the medium was changed to a medium containing 4-OH Tamoxifen (0.1, 1, 10, 20 μM) for 24 h.

### RNA Isolation and RT-PCR Analysis of ER Expression

Total cellular RNA was isolated from treated and untreated cells with RNeasy Mini Kit (Qiagen, Hilden, Germany). Using high-capacity cDNA archive kit (Applied Biosystems, Darmstadt, Germany) up to 1 μg total RNA was converted to cDNA. 2 μl of a 1: 5 dilution of the cDNA was used for the real-time PCR using a LightCycler (Roche, Heidelberg, Germany) real-time PCR analyzer and LightCycler cyber green reagents (Roche, Heidelberg, Germany). Each reaction contained 2.5 mM MgCl_2_. The thermal cycling conditions for 45 cycles were 92°C for 15 seconds, 60°C for 1 minute and 72°C for 30 seconds. Specific primers for ESR1 (5'- GCA CCC TGA AGT CTC TGG AA - 3', 5'- TGG CTA AAG TGG TGC ATG AT -3') and Actin (5'- CCA GCA CAA TGA AGA TCA AGA TC -3', 5'- ACA TCT GCT GGA AGG TGG ACA -3') were used. Linear amplification ranges were determined using LightCycler analysis software and reaction crossing points were applied to the formula described by Pfaffl [21].

### Cell Proliferation

For the determination of the cell count the cells were plated in medium at a concentration of 500,000 cells/100 mm tissue culture dishes. After 12 h, the cells were exposed to the indicated concentration of reagents for up to 132 h. At defined time points the processed cells were washed with PBS. After trypsinication the cells were resuspended in 5 ml fresh serum-containing medium and counted in a hemocytometer. For every time point three samples were counted in duplicates.

### Western Blot Analysis

MDA-MB-231 cells were plated in medium at a concentration of 500,000 cells/100 mm tissue culture dishes. One group of cells were pretreated with ribavirin (900 μM) and SAHA (10 μM) for 120+12 h while the other group remained untreated. Cells were then exposed to the indicated concentrations of 4-OH-Tam for 24 h, washed with PBS and lysed on ice in lysis buffer (100 mM Tris-HCl, pH 8.5, 100 mM NaCl, 0.5% NP40, 100 μg/ml PMSF). After lysis the cells were centrifuged at 13,000 rpm for 20 minutes at 4°C. Until usage the supernatants were stored at -20°C. Protein concentrations were quantified by absorption at 280 nm in 1:100 dilutions in 0.1 mM NaOH. 20 μg of total protein were analyzed under reducing conditions on a 12% polyacrylamide gel and blotted onto a nitrocellulose membrane. The blots were blocked over night in TBST buffer in 5% non-fat milk. For staining the blots were incubated with anti-human procaspase 8 polyclonal antibody (1:200), anti-human procaspase 9 monoclonal antibody (1:200) and anti-human Actin antibody (1:5000) followed by incubation with the peroxidase-conjugated anti-goat, anti-rabbit or anti-mouse antibodies (1:1000). Finally the blots were washed three times and then visualized with the ECL chemiluminescent detection system. The relative protein amount was normalized to Actin. Actin antibodies were obtained from Sigma-Aldrich (Heidelberg, Germany). Procaspase 8 and 9 antibodies were purchased from Santa Cruz (Heidelberg, Germany) (SC-6134 and SC-17784).

## Results

### Ribavirin-mediated Reactivation of Estrogen Receptor Gene Expression

**Figure 1:**
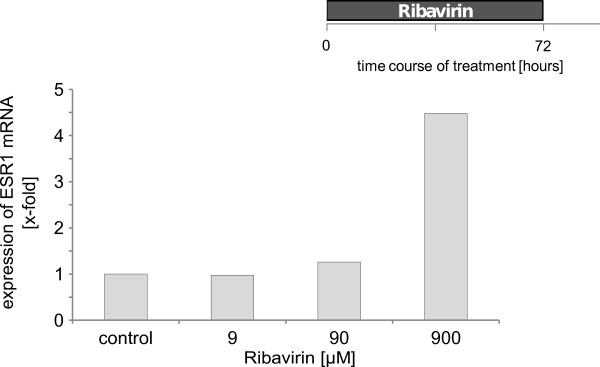
**Dose-dependent effect of ribavirin on ESR1 mRNA expression in MDA-MB-231 cells as measured by real-time PCR**. Cells were treated with different doses (9, 90 and 900 μM) of ribavirin for 72 h. The experiment has been done three times. Every experiment has shown an increase in expression of ESR1- however with a significant variation among the single experiments. A representative experiment is shown.

**Figure 2:**
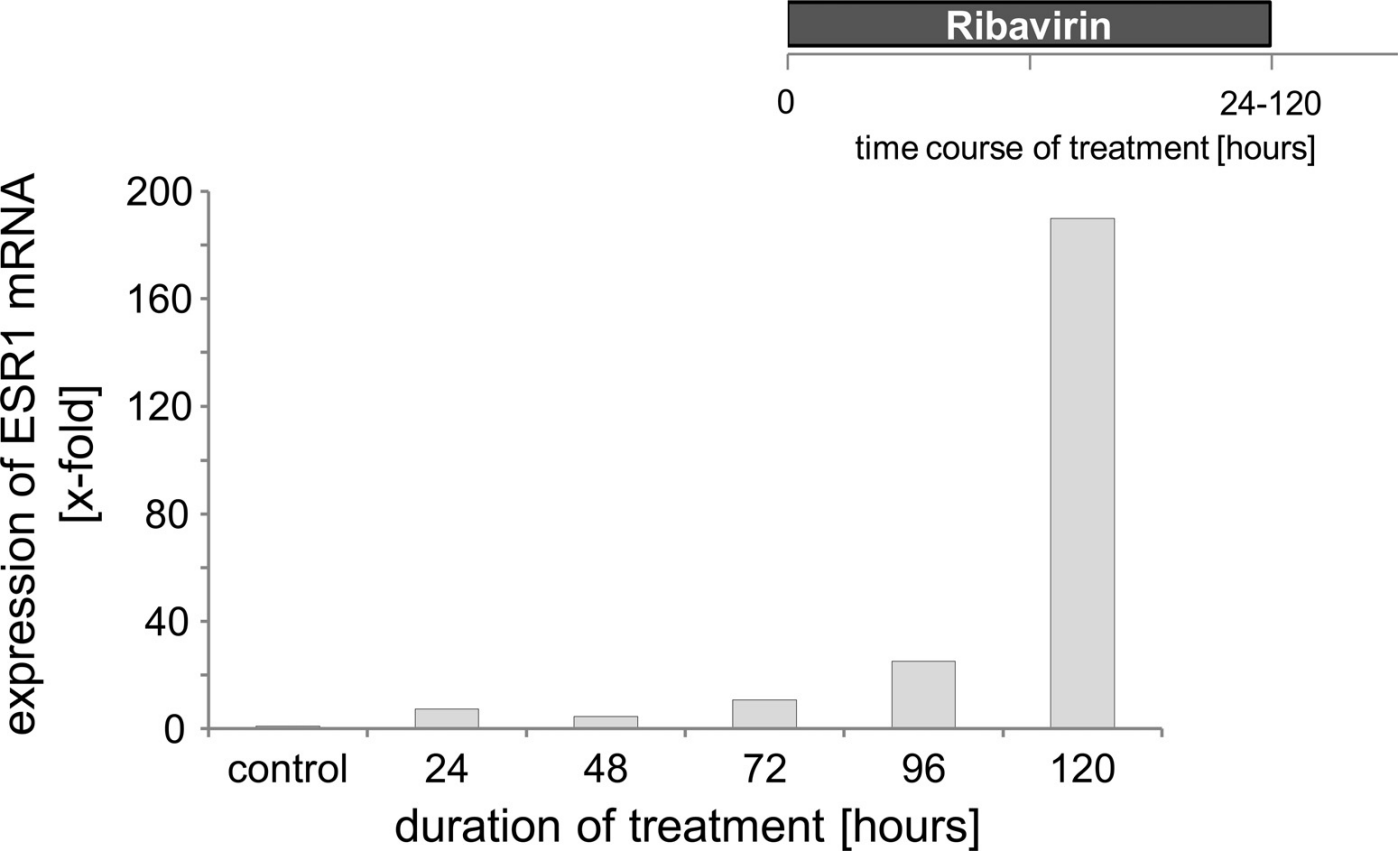
**Time-dependent effect of ribavirin on ESR1 mRNA expression in MDA-MB-231 cells as measured by real-time PCR**. Cells were treated with 900 μM ribavirin for up to 120 h (n = 3). Every experiment has shown a time-dependent increase in expression of ESR1- however with a significant variation among the single experiments. The experiment with the maximum effect is shown. The experiment with the minimum response is shown in supplemental material 1.

In order to explore the effect of Ribavirin on ESR gene expression, we used the ESR-negative cell line MDA-MB-231, since recent studies had shown a re-expression of ESR1 after treatment with hypomethylating agents [10]. We examined ESR1 expression by RT-PCR after drug exposure. The ESR-negative cell line was treated for 72 h with ribavirin at final concentrations of 9; 90 and 900 μM, respectively. A dose dependent increase in ESR1 mRNA was detected. The maximum ribavirin dose of 900 μM could not be further increased because of ribavirin cytotoxicity. At this dose level a 4.5 fold induction of ESR1 mRNA was reached [Figure 1]. In order to further determine whether this was already the peak expression level we studied the ESR1 gene expression over several time points for up to 120 h. The increase of ESR1 mRNA induction was time dependent with a maximum after 120 h. The increase of ESR1 mRNA induction was time dependent with a fold induction ranging from a minimum of 5.1 to a maximum of 190 (!) after 120 h [Figure 2 and additional file 1]. Since we were not certain whether we had already reached peak levels within 120 h, cells were treated for up to 8 days and ESR1 mRNA expression was measured every other day. In this series ESR1 mRNA expression was highest on day 6 with a 4.6 fold ESR1 expression vs. controls [data are not shown, additional file 2]. Along with earlier results, ribavirin induced ESR1 expression was identified to peak on day 5 at a final concentration of 900 μM.

In addition to the MDA-MB-231 cell line two ESR-negative cell lines were tested. Previous studies had identified ESR promoter hypermethylation in these two cell lines, UCCA-3199 and SK-BR-3 [22]. The ESR1 mRNA increase was determined after 5 days of exposure to 900 μM Ribavirin. Both cell lines responded well to ribavirin and while we observed only a minimum 1.5 fold induction of ESR1 expression in the UCCA-3199 cell line, a minimum 6.8 fold induction was identified in the SK-BR-3 cell line [cf. additional file 3].

**Figure 3:**
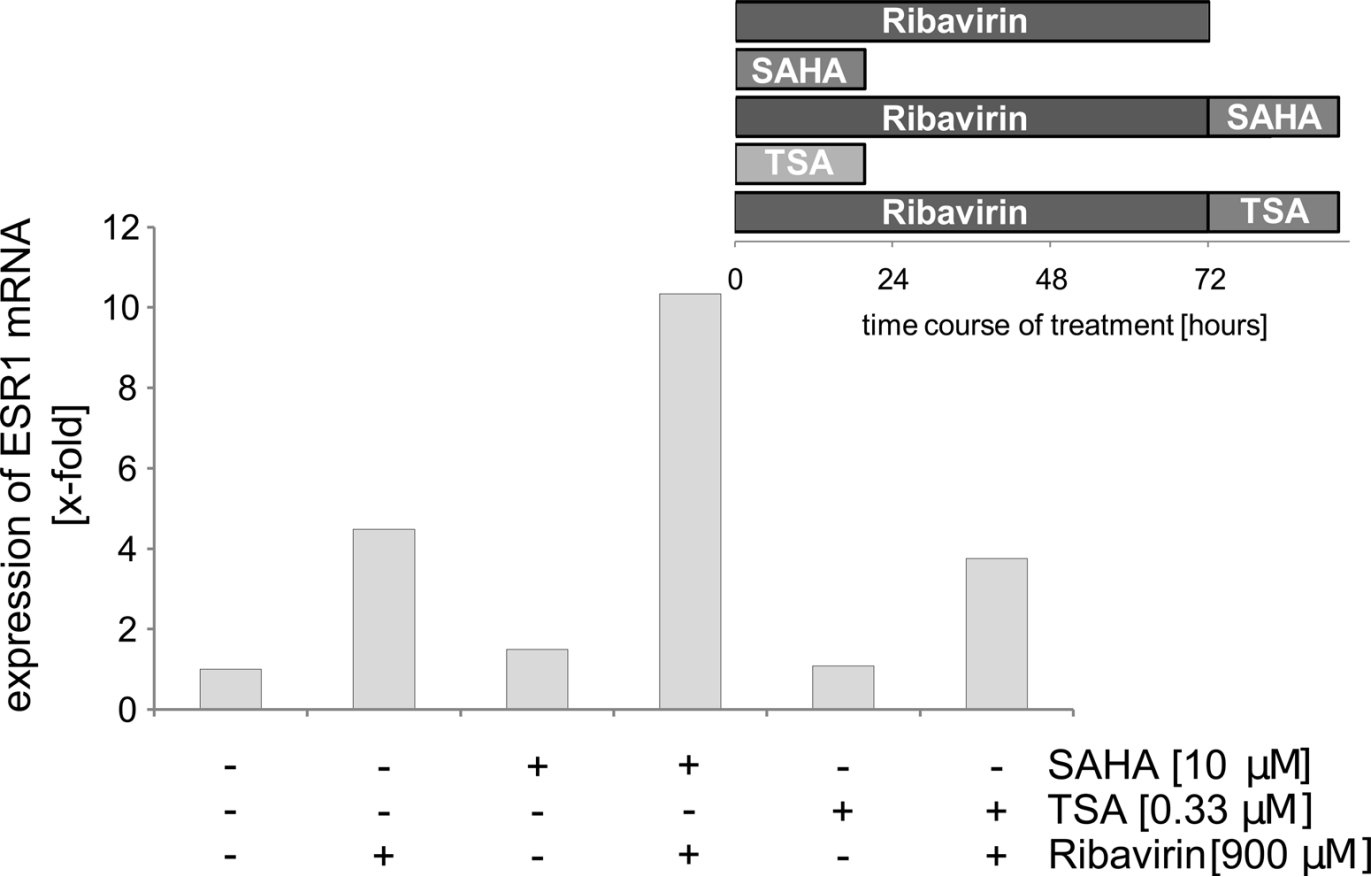
**The effect of ribavirin alone or in combination with TSA or SAHA on ESR1 mRNA expression in MDA-MB-231 cells as measured by real-time PCR**. Cells were treated with 900 μM ribavirin for 72 h alone or followed by 12 h treatment with 10 μM SAHA or 0.33 μM TSA (n = 3). Every experiment has shown the same effect as to the expression of ESR1 - however with a significant variation among the single experiments. A representative experiment is shown.

Previous studies have shown that the hypomethylating effect may be increased through combination of hypomethylating agents with an HDAC inhibitor [8]. In order to test whether the effect of ribavirin was also increasable in MDA-MB-231 cells, these were treated for 72 h with 900 μM ribavirin alone or followed by 12 h of treatment with 10 μM SAHA or 0.33 μM TSA. This condition has been shown as the optimal treatment scheme in previous studies [8]. After treatment the expression of ESR1 mRNA was measured. A minimum 10.3 fold induction was reached for the combination with SAHA and a minimum 3.8 fold induction for the combination with TSA [Figure 3]. After 120 h of treatment with 900 μM Ribavirin followed by 12 h treatment with 10 μM SAHA the increase of ESR1 mRNA induction reached a maximum 276 fold induction [cf. additional file 4]. In accordance with the literature, ESR1 immunostaining did unfortunately not give satisfactory results.

### Ribavirin Inhibits the Proliferation of MDA-MB-231 Cells

**Figure 4:**
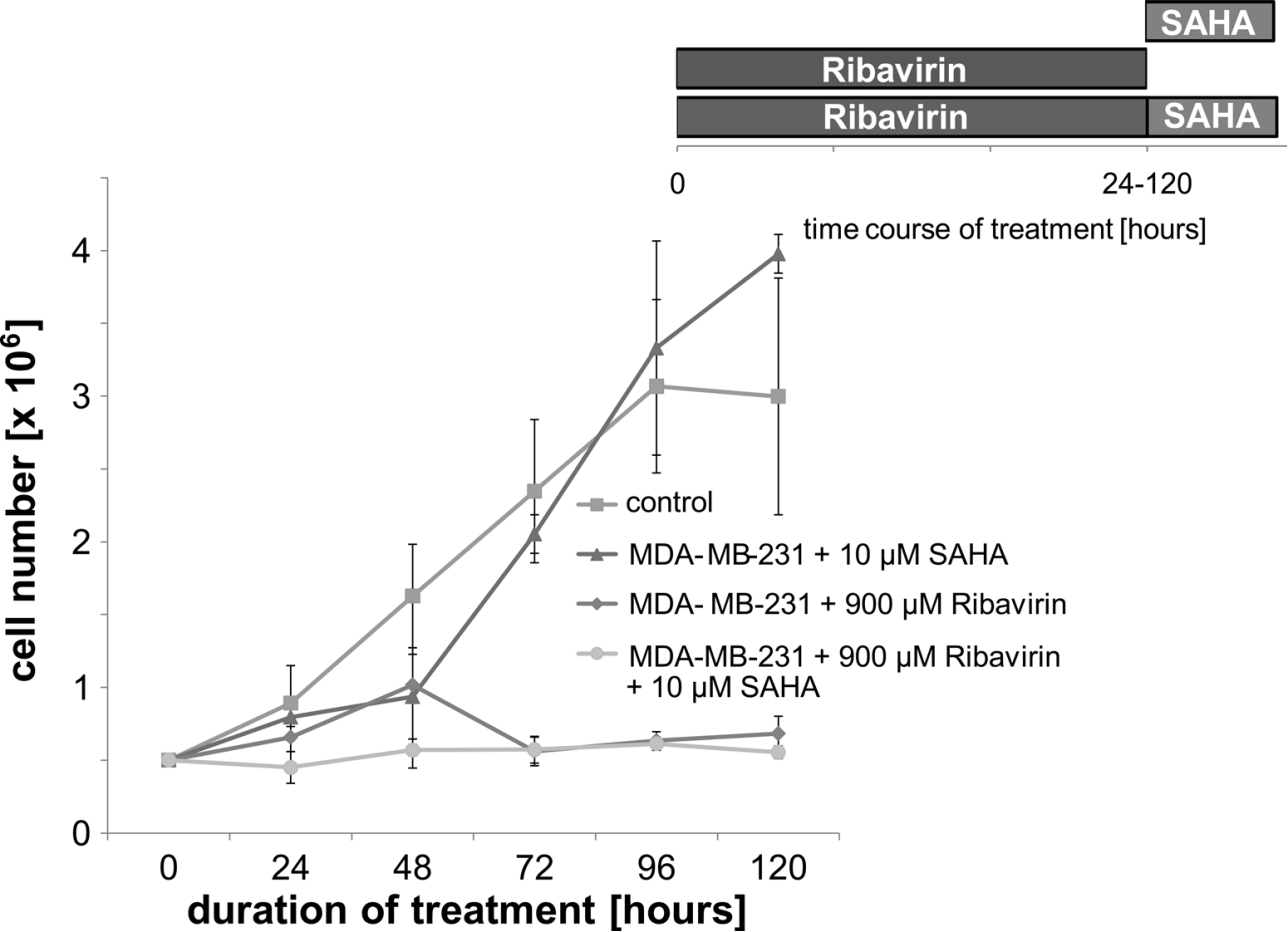
**The effect of ribavirin alone or in combination with the HDAC inhibitor SAHA in ESR1-negative breast cancer cells MDA-MB-231 on cell growth is shown**. The Figure shows the growth curves for untreated MDA-MB-231 cells vs. cells that were treated with 10 μM SAHA for 12 h or treated with 900 μM ribavirin for up to 120 h or treated with 900 μM ribavirin in combination with 10 μM SAHA for 12 h to 120 h. Each curve is the average of three independent experiments.

In order to determine the effect of ribavirin on MDA-MB-231 cell proliferation, cells were regularly counted on a hemocytometer during treatment with ribavirin. Cells were exposed to the following conditions: 900 μM ribavirin, 10 μM SAHA and 900 μM ribavirin plus 10 μM SAHA, respectively, counted at 24 h intervals and compared to the untreated controls. While SAHA alone had no effect on the cell proliferation, the treatment with ribavirin alone or in combination with SAHA inhibited cell proliferation. The calculation of the proliferation factor for ribavirin alone and in combination with SAHA resulted in a positive growing balance [Figure 4].

### Estrogen Receptor Gene Expression and Cell Growth Subsequent to Treatment with Ribavirin

**Figure 5:**
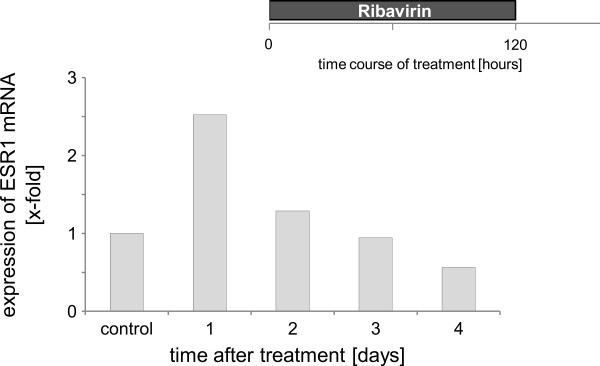
**The effect of ribavirin on ESR1 mRNA expression in MDA-MB-231 after treatment as measured by real-time PCR**. Cells were treated with 900 μM ribavirin for 120 h. mRNA was measured after treatment for up to 4 days (n = 3). Every experiment has shown the same effect as to the expression of ESR1 with a significant variation among the single experiments. A representative experiment is shown.

**Figure 6:**
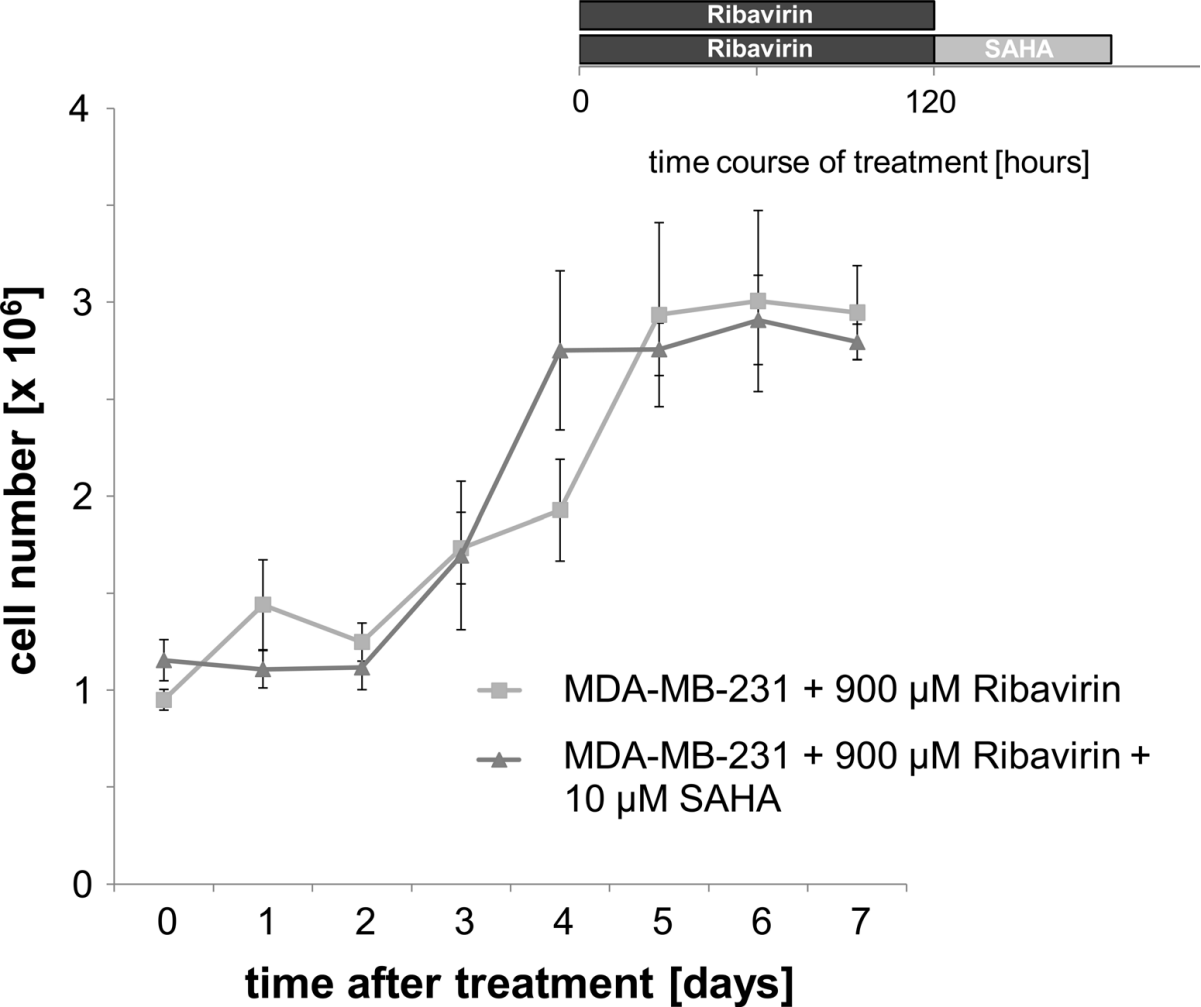
**The effect of ribavirin alone or in combination with SAHA in ESR1-negative breast cancer cells MDA-MB-231 on cell growth after treatment**. The figure demonstrates the growth curves for MDA-MB-231 cells after treatment with 900 μM ribavirin alone or in combination with 10 μM SAHA (120 h + 12 h). Cell numbers were measured after the treatment for up to 4 days. Each curve is the average of three independent experiments.

In order to determine the duration of ESR1 re-expression, cells were treated with 900 μM ribavirin for 5 days. Cell culture medium was changed and cell samples were analyzed on a daily basis. ESR1 mRNA levels were quantified in relation to untreated cells. The re-expression of ESR1 mRNA decreased during the observed treatment course [Figure 5] and reached ESR1 mRNA levels of untreated cells 72 h after treatment discontinuation. Based on a potentially altered cell growth behavior under treatment, the cell growth was further determined for up to 5 days after treatment with 900 μM ribavirin and in one treatment arm it was followed by a 12 h exposure to 10 μM SAHA. After 72 h of treatment the effect of ribavirin on cell growth was no longer present. The determination of the growth curve and the proliferation factor showed the same pattern as for untreated cells, which allows the conclusion that the effect of ribavirin in the breast cancer cells that have been studied is reversible[Figure 6].

### Ribavirin Induces a Tamoxifen Sensitive Expression of ESR

**Figure 7:**
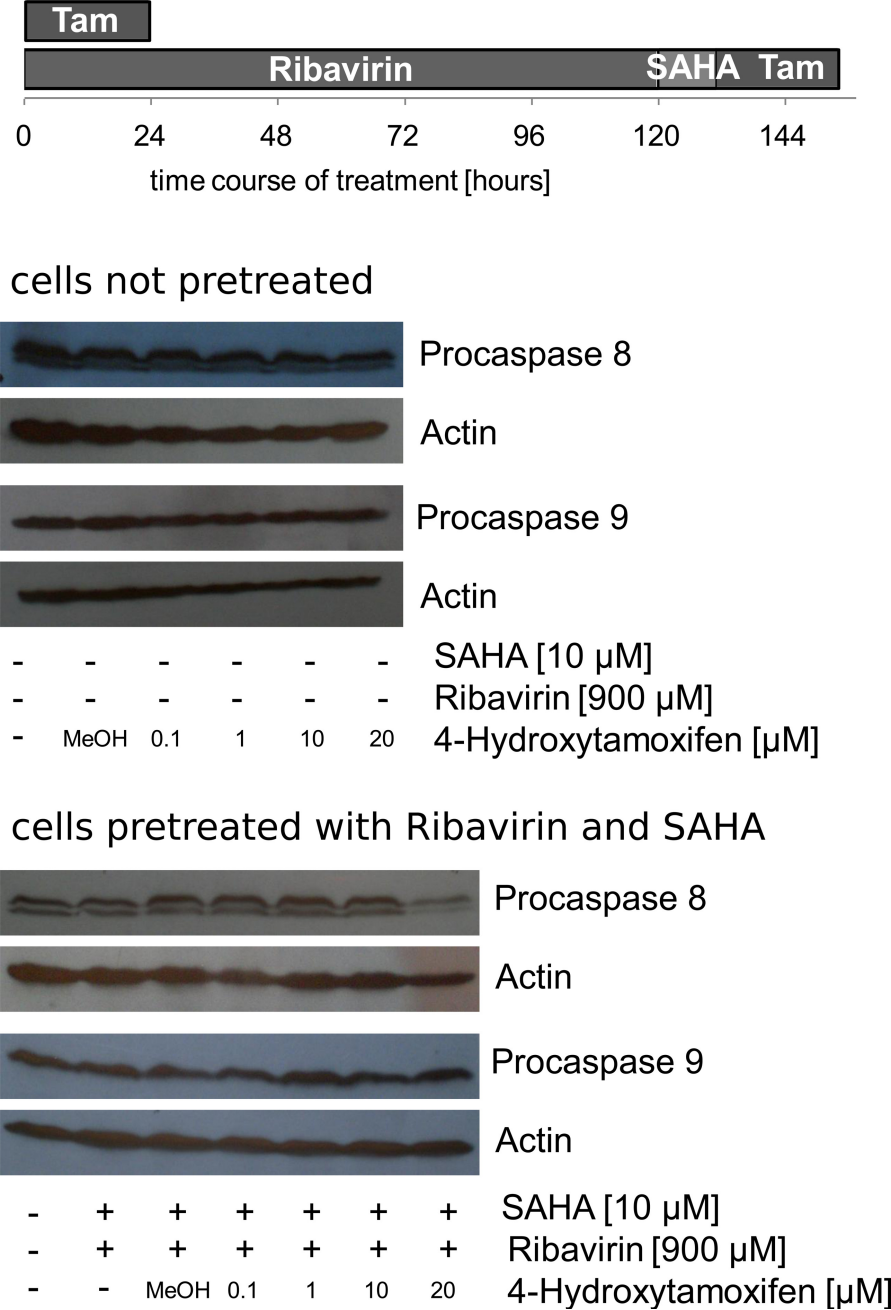
**Effect of ribavirin in combination with SAHA in ESR1-negative breast cancer cells MDA-MB-231 on tamoxifen sensitivity**. Western blot analyses of procaspase 8 and 9 are shown after 24 h of treatment with different concentrations of 4-OHTam. They were either pretreated with 900 μM ribavirin in combination with 10 μM SAHA or remained untreated MDA-MB-231 cells. β-Actin was probed as the loading control. The decrease of procaspase levels is an indication of increased apoptosis, which is being caused by pretreatment with ribavirin and SAHA. A representative experiment is shown.

Our study identified ESR expression to peak after 5 days of treatment with 900 μM ribavirin followed by 12 h of treatment with 10 μM SAHA. This effect was detectable for up to 48 h after treatment discontinuation. In order to further assess the expression of a functional ESR1 protein, cells were exposed to 4-hydroxytamoxifen (4-OH-Tam) at different concentrations for 24 h subsequent to treatment with ribavirin plus SAHA. Apoptosis was measured by determination of procaspase-8 and the procaspase-9 levels. To prevent potential interactions between the agents that were used, the cell culture medium was changed prior to treatment with 4-OH-Tam. Cells that were treated with the combination of ribavirin plus SAHA experienced a decrease of procaspase 8 levels subsequent to treatment with 20 μM 4-OH-Tam, which indicated increased apoptosis in the presence of 4-OH-Tam subsequent to re-expression of a functional ESR protein which sensitized the cells to 4-OH-Tam treatment [Figure 7].

## Discussion

In this report we demonstrate ribavirin to induce expression of the ESR1 both, on the RNA and the functional levels.

The presence of the ESR is an important prognostic indicator as to the responsiveness of breast cancer to an endocrine therapy. The lack of ESR expression (more precisely ESR1) is the predominant mechanism that has been ascribed to *de novo *endocrine treatment resistance [4]. In less than 1% the loss of ESR1 expression results from genetic mutations [7]. In addition, earlier studies have shown that the absence of ESR may also be the result of aberrant epigenetic modifications, such as the deacetylation of histones and methylation of DNA at the level of the ESR promoter [23]. The treatment of ESR-negative breast cancer cells with hypomethylating agents, such as 5-azacytidine or 5-aza-2'-deoxycytidine has been reported to go along with the re-expression of functionally active ESR [10]. 5-aza-2'-deoxycytidine induces the re-expression of ESR by inhibition of the DNA methyltransferase (DNMT) through irreversible binding of the DNMT [11].

In the study presented herein, we show ribavirin to induce the re-expression of functionally active ESR1 in ESR-negative breast cancer cells.

Based on structural similarities with 5-azycytidine, ribavirin was chosen in order to assess for potential hypomethylating functional activities on ESR1 expression. Ribavirin is a known inhibitor of the S-adenosyl-L-homocysteine (SAH) hydrolase [19]. Inhibition of the SAH hydrolase goes along with increased levels of SAH, which in turn, is a potent inhibitor of the DNMT [17]. Our data show a dose dependent up regulation of ESR1 at the mRNA level upon treatment with ribavirin [Figure 1], which peaks at concentrations of 900 μM. This *in vitro *concentration equals to the 100-fold concentration of the mean steady state plasma concentration after four weeks of treatment with 600 mg ribavirin being administered twice daily [24].

This increase is also time dependent with a peak on day 5 [Figure 2 and additional files].

Numerous studies have shown previously that the effect of hypomethylating agents such as 5-aza-2'-deoxycytidine may be potentiated by co-treatment with an HDAC inhibitor (e.g. TSA or SAHA) [8,25]. In this study we explored whether the combination of ribavirin with TSA or SAHA would go along with similar effects. The treatment agents were administered sequentially: the cells were treated with 900 μM ribavirin over 3 days first, which was followed by a 12 h treatment with 10 μM SAHA or 0.33 μM TSA, which resulted in an up regulation of the ESR1 mRNA expression. This effect was more pronounced for SAHA than for TSA and suggested a synergistic effect for the combination of ribavirin with HDAC inhibitors. Similar synergistic effects have also been observed for combinations of 5-azacytidine with HDAC inhibitors [Figure 3].

Several studies have reported that both, the hypomethylation through 5-aza-2'-deoxycytidine and HDAC inhibition through TSA inhibit the growth of breast cancer cells either alone or in combination [26-28]. We observed that treatment with ribavirin alone or in combination with SAHA inhibits the growth of the breast cancer cells, while SAHA alone has no effect. Ribavirin alone or in combination with TSA had similar effects [Figure 4].

Since earlier studies had pointed out that the effect of 5-azacytidine was the result from an irreversible binding to the DNMT [29,30]. We were wondering whether this was also true for ribavirin. 48 h after the end of the treatment with ribavirin or ribavirin in combination with SAHA the cells grew just like the untreated controls and we were able to observe a decrease in the ESR1 mRNA expression [Figures 5, 6], which led us to the conclusion that the effect of ribavirin was reversible.

In order to further explore whether the re-expressed ESR was functionally active, we treated our cells with 4-hydroxytamoxifen (4-OHTam), which is the active metabolite of tamoxifen and observed an adequate response to endocrine therapy, which was in accordance with earlier studies, which showed the sensitization of estrogen-negative breast cancer cells to endocrine therapy after treatment with 5-aza-2'-deoxycytidine in combination with TSA [28]. In cells that were pretreated with a combination of ribavirin plus SAHA we were able to see an induction of apoptosis at concentration of 20 μM 4-OHTam, as assessed by adecreased level of procaspase-8. The data presented herein indicate that MDA-MB-231 cells re-express a functionally active ESR1 subsequent to treatment with Ribavirin and SAHA [Figure 7].

In summary, this study leads us to the conclusion that that functionally active ESR1 can be re-expressed in estrogen-negative breast cancer cells by treatment with ribavirin and SAHA, which restores the sensitivity to 4-OHTam endocrine treatment. Ribavirin and analogs could therefore pave the way to the development of novel therapeutic strategies, which aim at the re-expression of ESR in order to restore the susceptibility to tamoxifen-related treatment regimens.

## Abbreviations

ESR1: estrogen receptor alpha; HDAC: histone deacetylase; DNMT: DNA methyltransferase; SAHA: suberoylanilide hydroxamic acid; TSA: Trichostatin A; 4-OHTam: 4-hydroxytamoxifen; SAH: S-adenosylhomocysteine; SAM: S-adenosylmethionine.

## Competing interests

The authors declare that they have no conflict of interest or any financial relationship with the work presented herein.

## Authors' contributions

AS carried out the molecular genetic analyses, the cytological analyses, the immunoassays and performed the statistical analysis. UM planned the study. Both authors worked on the manuscript. All authors read and approved the final manuscript.

## Supplementary Material

Additional file 1 **Time-dependent effect of ribavirin on ESR1 mRNA expression in MDA-MB-231 cells as measured by real-time PCR**. Cells were treated with 900 μM ribavirin for up to 120 h. The experiment with the minimum effect is shown. Click here for file

Additional file 2 **The time-dependent effect of ribavirin on ESR1 mRNA expression in MDA-MB-231 cells as measured by real-time PCR**. Cells were treated with 900 μM ribavirin for up to 8 days. The maximum effect on ESR1 mRNA was observed on day 6 (n = 3). Every experiment has shown a time-dependent increase in expression of ESR1. The results varied significantly. A representative experiment is shown. Click here for file

Additional file 3 **Effect of ribavirin on different ESR1 negative breast cancer cells as measured by real-time PCR**. MDA-MB-231, UACC-3199 and SK-BR-3 cells were treated with 900 μM ribavirin for 5 days. A representative example of two experiments that yielded similar results is shown. Click here for file

Additional file 4 **Time-dependent effect of ribavirin in combination with SAHA on ESR1 mRNA expression in MDA-MB-231 cells as measured by real-time PCR**. Cells were treated with 900 μM of ribavirin for up to 120 h followed by a 12 h treatment with 10 μM SAHA (n = 3). Every experiment has shown a time-dependent increase as to the expression of ESR1. The experiments varied strongly. The experiment with the maximum effect is shown. Click here for file
