# Supplementary figures and images for: Ribavirin restores ESR1 gene expression and tamoxifen sensitivity in ESR1 negative breast cancer cell lines
Source: Clin Epigenetics. 2011 Dec 5;3(1):8. doi: 10.1186/1868-7083-3-8 (PMC3305339; doi:10.1186/1868-7083-3-8)

expression of ESR1 mRNA  
[x-fold]

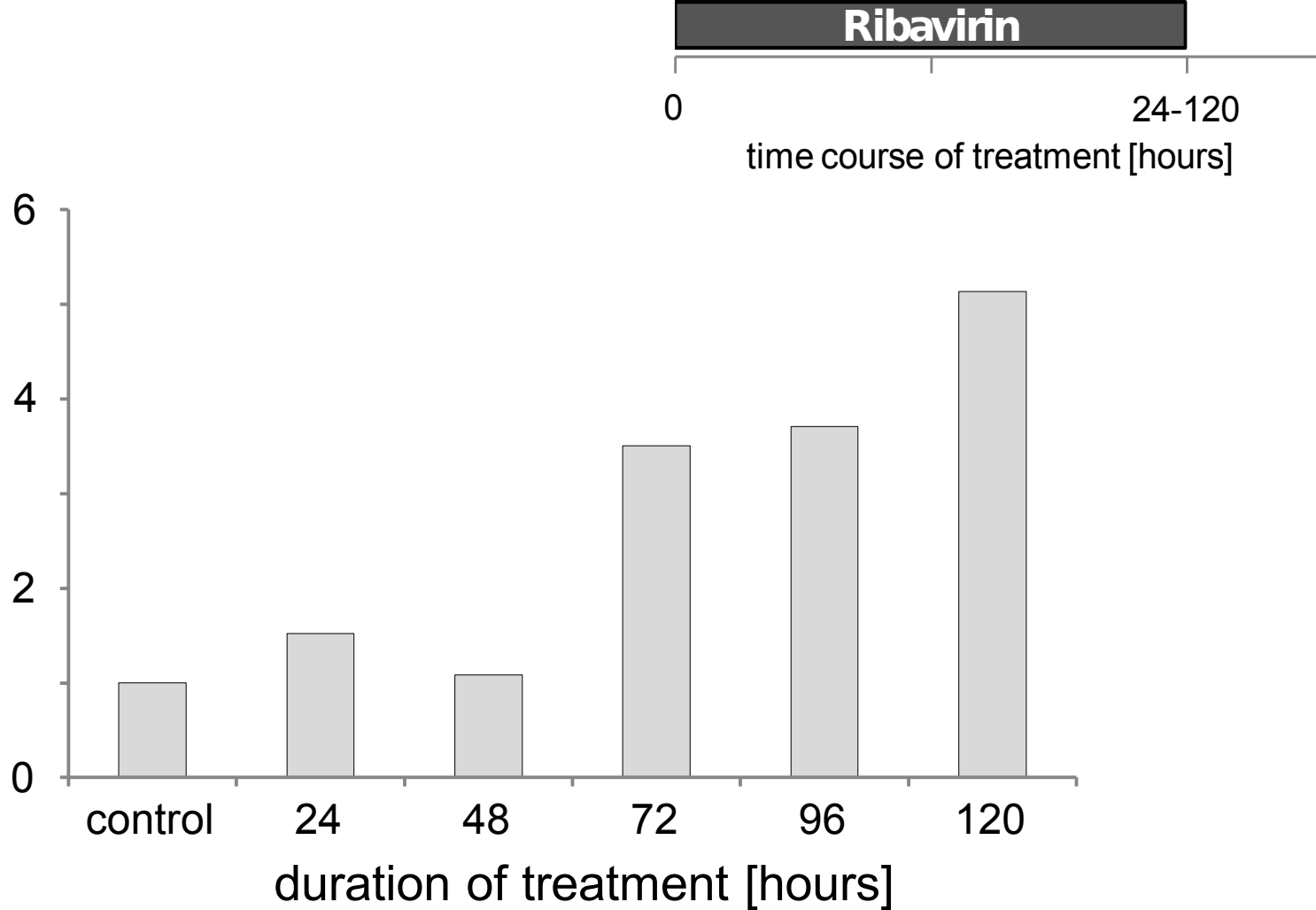

Supplement: Additional file 1 — Time-dependent effect of ribavirin on ESR1 mRNA expression in MDA-MB-231 cells as measured by real-time PCR. Cells were treated with 900 μM ribavirin for up to 120 h. The experiment with the minimum effect is shown. [file 1868-7083-3-8-S1.PDF]

expression of ESR1 mRNA  
[x-fold]

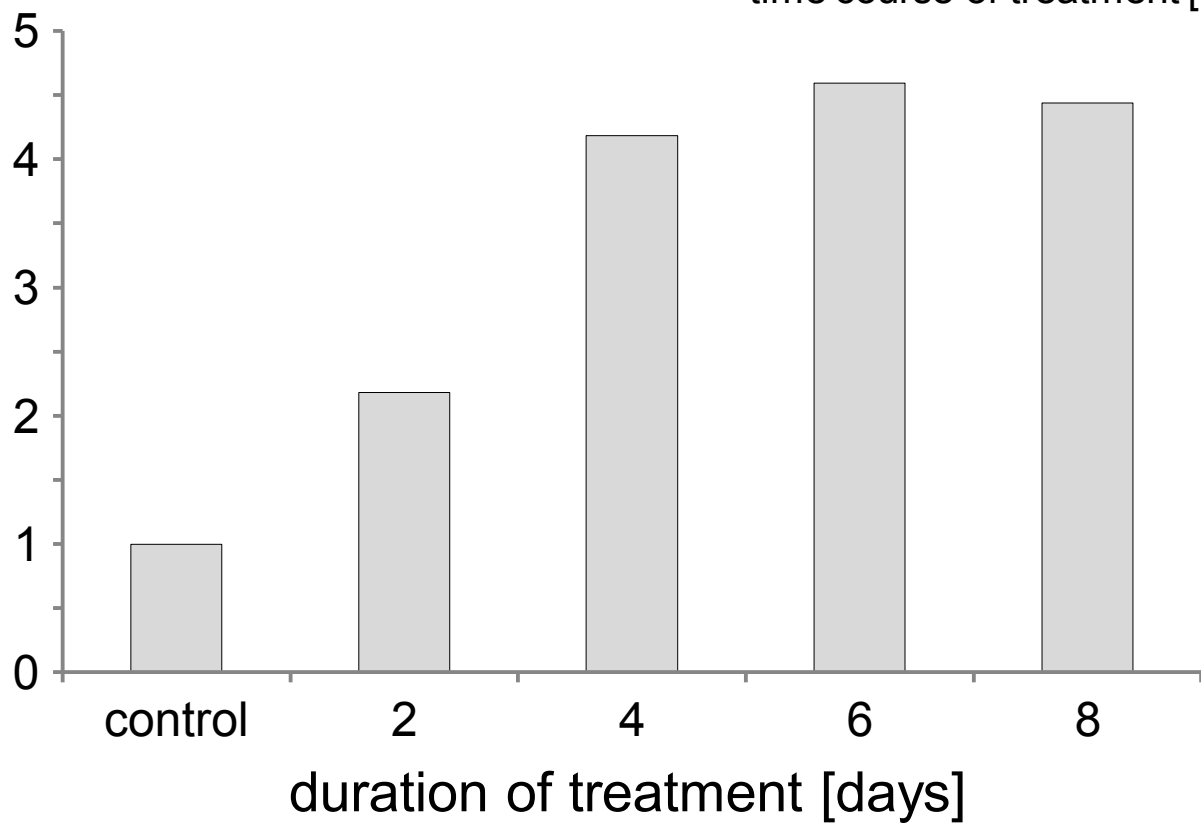

**Ribavirin**

0

2-8

time course of treatment [days]

Supplement: Additional file 2 — The time-dependent effect of ribavirin on ESR1 mRNA expression in MDA-MB-231 cells as measured by real-time PCR. Cells were treated with 900 μM ribavirin for up to 8 days. The maximum effect on ESR1 mRNA was observed on day 6 (n = 3). Every experiment has shown a time-dependent increase in expression of ESR1. The results varied significantly. A representative experiment is shown. [file 1868-7083-3-8-S2.PDF]

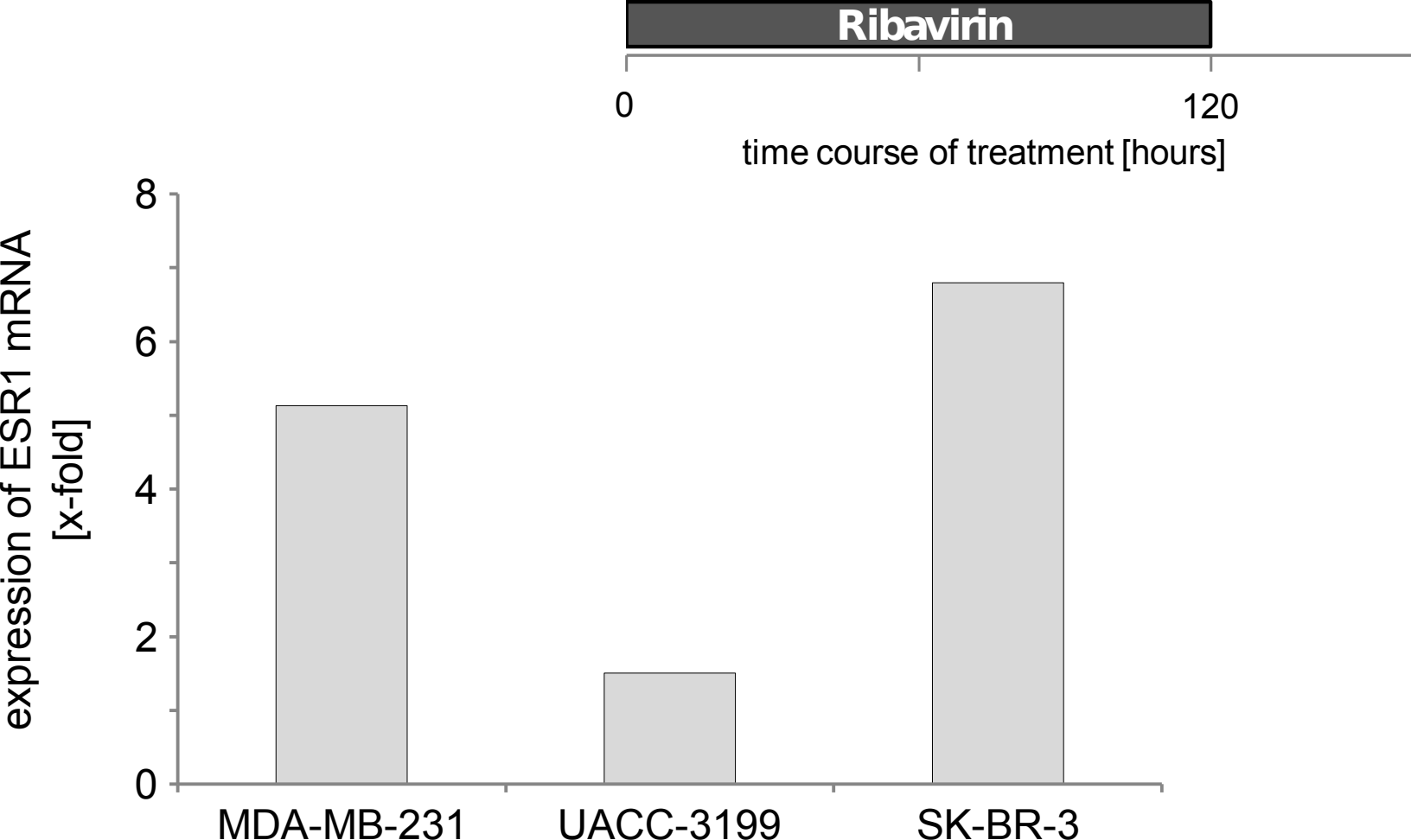

Supplement: Additional file 3 — Effect of ribavirin on different ESR1 negative breast cancer cells as measured by real-time PCR. MDA-MB-231, UACC-3199 and SK-BR-3 cells were treated with 900 μM ribavirin for 5 days. A representative example of two experiments that yielded similar results is shown. [file 1868-7083-3-8-S3.PDF]
