# Supplementary material for: Ribavirin restores ESR1 gene expression and tamoxifen sensitivity in ESR1 negative breast cancer cell lines
Source: Clin Epigenetics. 2011 Dec 5;3(1):8. doi: 10.1186/1868-7083-3-8 (PMC3305339; doi:10.1186/1868-7083-3-8)
Supplement: Additional file 4 — Time-dependent effect of ribavirin in combination with SAHA on ESR1 mRNA expression in MDA-MB-231 cells as measured by real-time PCR. Cells were treated with 900 μM of ribavirin for up to 120 h followed by a 12 h treatment with 10 μM SAHA (n = 3). Every experiment has shown a time-dependent increase as to the expression of ESR1. The experiments varied strongly. The experiment with the maximum effect is shown. [file 1868-7083-3-8-S4.PDF]

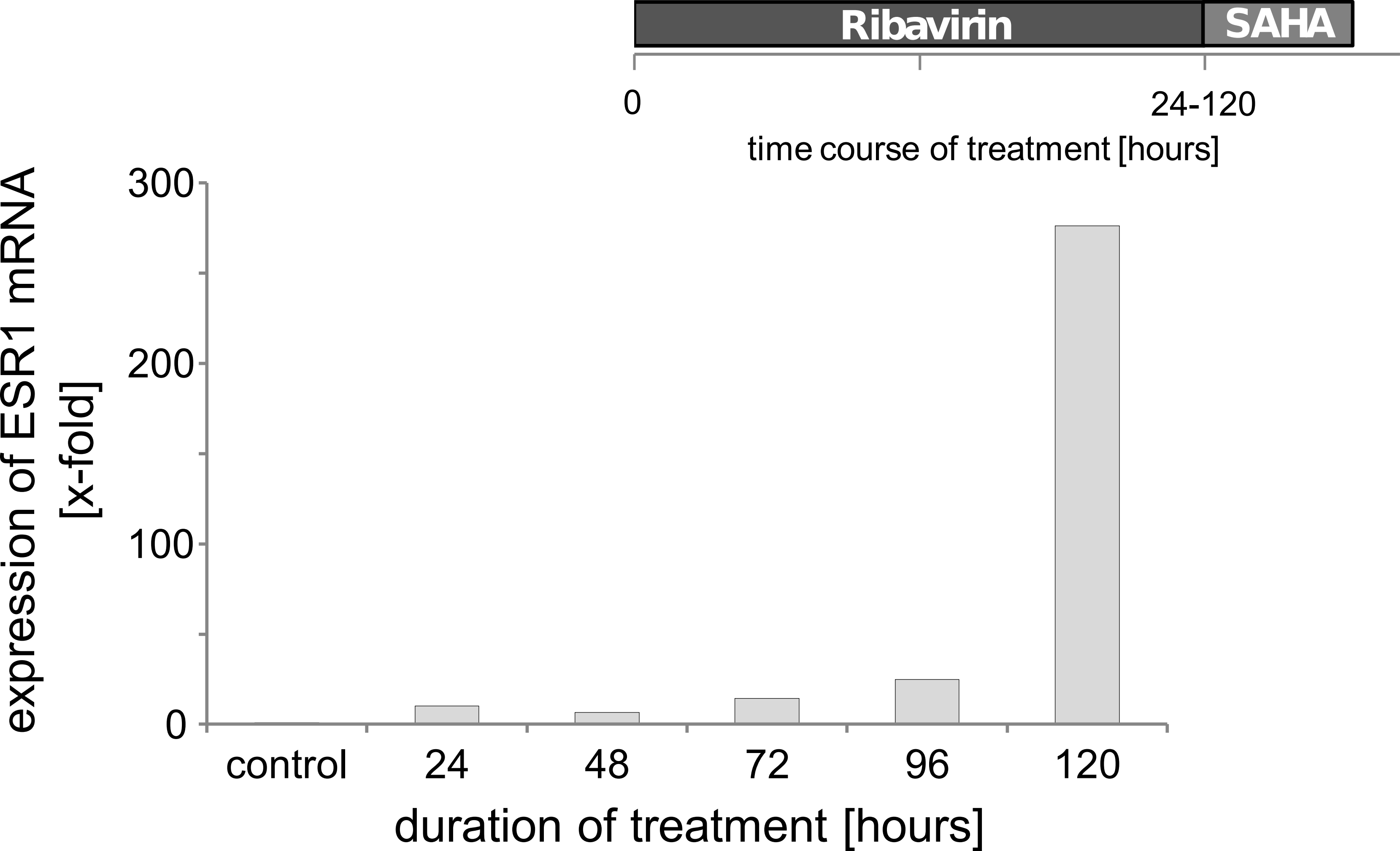

Ribavirin
SAHA
0
24-120
time course of treatment [hours]
expression of ESR1 mRNA [x-fold]
300
200
100
0
control
24
48
72
96
120
duration of treatment [hours]
